# A Case of Fulminant Meningococcemia: It Is All in the Complement

**DOI:** 10.1155/2017/6093695

**Published:** 2017-07-20

**Authors:** Kellie L. Hawkins, Mariah Hoffman, Sonia Okuyama, Sarah E. Rowan

**Affiliations:** ^1^Division of Infectious Disease, Department of Medicine, University of Colorado School of Medicine, Aurora, CO, USA; ^2^Denver Health and Hospital Authority, Denver, CO, USA; ^3^Division of Internal Medicine, Department of Medicine, University of Colorado School of Medicine, Aurora, CO, USA; ^4^Division of Oncology, Department of Medicine, University of Colorado School of Medicine, Aurora, CO, USA

## Abstract

Eculizumab is a novel monoclonal antibody that inhibits complement-mediated hemolysis in patients with paroxysmal nocturnal hemoglobinuria (PNH). Complement deficiency is a well-known risk factor for meningococcal infection. We describe a case of a young patient with PNH treated with eculizumab who presented with a life-threatening case of nongroupable meningococcemia. As this new biologic agent becomes more widely prescribed, providers should be aware of the increased risk of meningococcemia. In addition to vaccination, providers may consider the use of oral penicillin for antibiotic prophylaxis against* Neisseria meningitidis* in these cases of functional complement deficiency.

## 1. Case Presentation

An 18-year-old female with paroxysmal nocturnal hemoglobinuria (PNH) presented to the emergency department with one day of progressively worsening malaise, nausea, vomiting, diarrhea, fever, and rash. The patient's only significant past medical history was PNH, which was diagnosed two years prior to presentation in the setting of primary Budd-Chiari syndrome caused by hepatic vein thrombosis. She was also noted to be anemic at that time. The hepatic vein thrombosis was treated with a transjugular intrahepatic portosystemic shunt (TIPS) and anticoagulation with warfarin was initiated as secondary prevention for further thrombosis. In addition to warfarin, her other medications included eculizumab (initiated upon diagnosis of PNH), vitamin B12, and folate. The MenACWY-D conjugated vaccine was given prior to receipt of eculizumab. The vaccine against serogroup B was not yet approved for market use.

**Figure 1:**
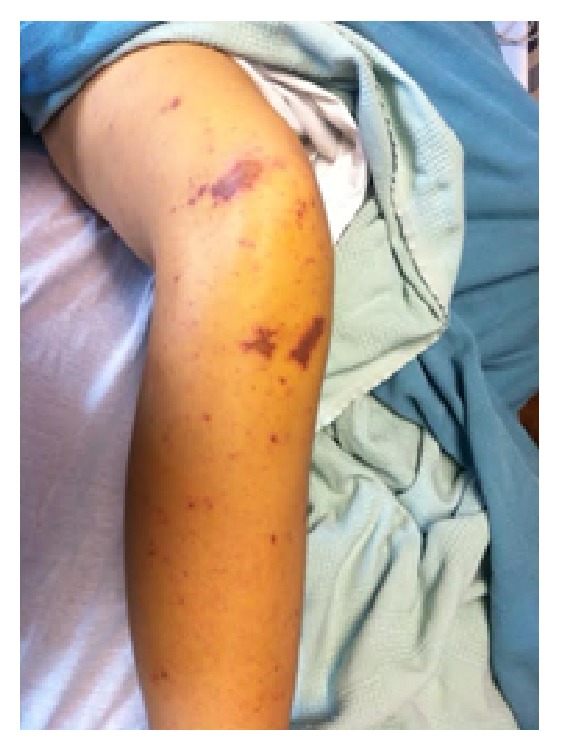
Right lower extremity with purpura, photo cred M. Hoffman 2015.

**Figure 2:**
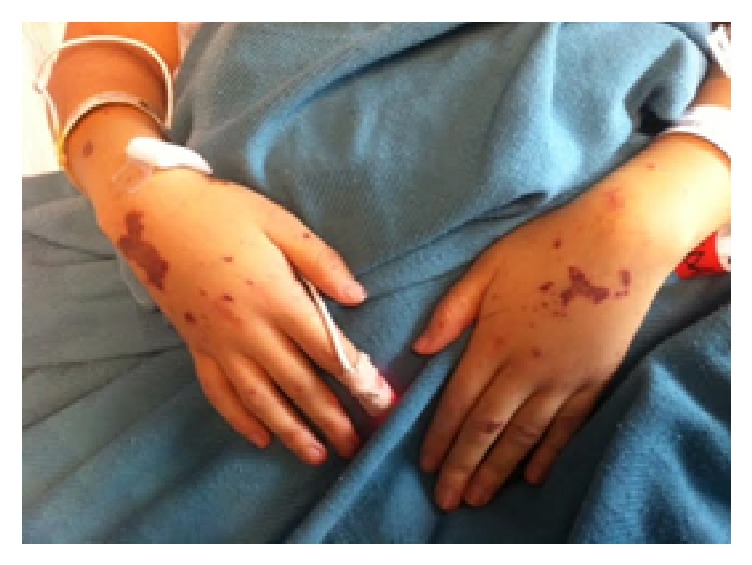
Bilateral upper extremities with purpura, photo cred M. Hoffman 2015.

**Figure 3:**
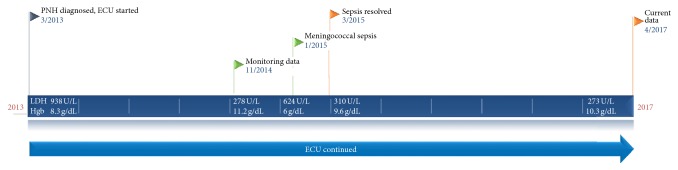
Timeline of events. Dates and events are noted next to flags. Corresponding laboratory values are noted in the rectangular boxes. PNH: paroxysmal nocturnal hemoglobinuria, ECU: eculizumab, LDH: lactate dehydrogenase, and Hgb: hemoglobin.

Upon presentation to the emergency department, the patient was noted to be lethargic and hypotensive with a diffuse purpuric rash. Her initial vital signs were consistent with distributive shock: blood pressure 63/39 mmHg, heart rate 129 bpm, respiratory rate 35 bpm, and oxygen saturation 95%. Pertinent physical findings included bilateral conjunctival hemorrhages, jaundice, and diffuse purpura (Figures 1 and 2). She had a left subclavian port that was clean with no surrounding erythema, exudate, or induration. Laboratory values were notable for white blood cell count of 18.1 k/*μ*L, platelets 67  k/*μ*L, lactate 8.9 mmol/L, total bilirubin 21.8 mg/dL (direct 10.7 mg/dL), haptoglobin <7 mg/dL (reference range 20–135 mg/dL), and an INR of 5.18 (reference range 0.33–1.19). See Figure 3. An abdominal ultrasound with Doppler revealed patent TIPS and patent hepatic vasculature including the hepatic vein.

The patient was immediately intubated for airway protection. Resuscitation was initiated with intravenous fluids and vasopressors. Her blood pressure remained low despite maximum doses of vasopressors. Intravenous antibiotics were administered including vancomycin, cefepime, and ampicillin. Hydrocortisone was administered as adjunctive therapy in the setting of sepsis and concern for Waterhouse-Friderichsen syndrome. She was admitted to the intensive care unit for further management. There her hemodynamics stabilized twenty-four hours after her initial presentation. Blood cultures grew Gram-negative diplococci identified as* Neisseria meningitidis*. The bacterial isolate was sent to the state health department laboratory for serogrouping and was determined to be nongroupable. Upon confirming* Neisseria meningitidis *infection, a contact investigation was launched by the hospital epidemiology team to identify those requiring meningococcal prophylaxis.

After* Neisseria meningitidis *was identified, the antibiotics were narrowed to ceftriaxone 2 grams every 12 hours and then later switched to cefotaxime 2 grams every 6 hours to avoid potential hepatic toxicity. High dose steroids were carefully tapered off and the patient displayed no signs of Waterhouse-Friderichsen syndrome. Eculizumab dosing was continued at her normal dosing interval. Intravenous antibiotics were continued for a total of 14 days, after which she began taking penicillin-VK 250 mg orally twice daily to be continued indefinitely for* Neisseria meningitidis* prophylaxis. Once available, she also received the vaccine against* Neisseria meningitidis* serogroup B. She will require a booster of the conjugated MenACWY vaccine every 5 years. She was given a prescription for oral ciprofloxacin at a dose of 750 mg to have on hand for “rescue therapy” if symptoms of meningococcal infection recur, with strict recommendations to also seek immediate medical attention in this circumstance. Fortunately, the patient has done very well since this life-threatening event with no further infectious issues. Her TIPS continues to function well and she has normal liver function. She continues to have evidence of mild hemolysis (slightly elevated bilirubin and LDH) on eculizumab but overall her symptoms are controlled.

## 2. Discussion

PNH is a rare disease. The acquired mutation in PNH is in the PIGA gene and originates in hematopoietic stem cells. The mutation causes downstream effects on anchoring proteins, including those that anchor proteins involved with the terminal complement system. This mutation makes red blood cells susceptible to activated complement and hemolysis [1]. Eculizumab is a novel monoclonal antibody therapy for PNH that inhibits the terminal complement system, preventing PNH-associated hemolysis and thrombosis. The monoclonal antibody works by binding to C5 and inhibiting activation of the terminal complement system. Eculizumab induces a “functional complement deficiency” and increases the risk of infection with encapsulated bacteria especially* Neisseria meningitidis. * Eculizumab is the only pharmaceutical agent approved for paroxysmal nocturnal hemoglobinuria and is very effective at reducing the hemolysis and thromboembolic events associated with this disease, though rare breakthrough embolic events are reported. This agent is also used in atypical hemolytic uremic syndrome [2, 3].

Eculizumab has a black box warning regarding the increased risk of* Neisseria meningitidis* infection because it makes the host functionally complement deficient. The reported occurrence of meningococcal infection with use of eculizumab is approximately 0.5% per 100 patient years [4, 5]. The medication is dispensed under a restricted program and providers must comply with recommendations from the Advisory Committee on Immunization Practices (ACIP) [6]. For patients on eculizumab, current practice guidelines suggest administering two doses of conjugated MenACWY vaccine at least two months apart with revaccination every five years [7]. It is now recommended to also administer the MenB vaccine series [8]. There are no official recommendations regarding revaccination with MenB but a booster dose is currently under consideration from the ACIP as data becomes available [4]. Additionally, there are no current guidelines regarding the use of prophylactic oral penicillin; however many experts recommend this practice for patients on eculizumab. Lastly, all patients are supplied with a safety card from the manufacturer of eculizumab that describes signs and symptoms of meningococcal disease and when to seek medical attention [5].

This case presentation highlights a life-threatening case of meningococcal disease complicated by purpura fulminans. As of November 2014, there were sixteen reported cases of meningococcal infections associated with the use of eculizumab in the United States and this case would be the seventeenth [5, 9–12]. Our patient acquired a nongroupable strain of meningococcal disease while on eculizumab. She was not protected from her prior vaccination as MenACWY vaccine would not be expected to provide protection against nongroupable or other serogroups of meningococci. She was not on prophylactic oral penicillin prior to becoming ill. It is of critical importance that both primary care physicians and specialists take care to ensure people on eculizumab are properly vaccinated. In addition to vaccination, some providers have measured response titers to vaccination as a correlate of protection [13]. Further, patients and providers should strongly consider the use of oral penicillin for antibiotic prophylaxis against* Neisseria meningitidis* for patients taking eculizumab.

## References

[1] Takeda J., Miyata T., Kawagoe K. (1993). Deficiency of the GPI anchor caused by a somatic mutation of the PIG-A gene in paroxysmal nocturnal hemoglobinuria. *Cell*.

[2] Kelly R., Richards S., Hillmen P., Hill A. (2009). The pathophysiology of paroxysmal nocturnal hemoglobinuria and treatment with eculizumab. *Therapeutics and Clinical Risk Management*.

[3] Hillmen P., Young N. S., Schubert J. (2006). The complement inhibitor eculizumab in paroxysmal nocturnal hemoglobinuria. *The New England Journal of Medicine*.

[4] Patton M. E., Stephens D., Moore K., MacNeil J. R. (2017). Updated Recommendations for Use of MenB-FHbp Serogroup B Meningococcal Vaccine — Advisory Committee on Immunization Practices, 2016. *MMWR. Morbidity and Mortality Weekly Report*.

[5] FDA Drug Safety and Risk Management Advisory Committee. Soliris® (eculizumab). https://www.fda.gov/downloads/AdvisoryCommittees/CommitteesMeetingMaterials/Drugs/DrugSafetyandRiskManagementAdvisoryCommittee/UCM426664.pdf.

[6] Bouts A., Monnens L., Davin J.-C., Struijk G., Spanjaard L. (2011). Insufficient protection by Neisseria meningitidis vaccination alone during eculizumab therapy. *Pediatric Nephrology*.

[7] Brodsky R. A., Young N. S., Antonioli E. (2008). Multicenter phase 3 study of the complement inhibitor eculizumab for the treatment of patients with paroxysmal nocturnal hemoglobinuria. *Blood*.

[8] Cohn A. C., MacNeil J. R., Clark T. A. (2013). Centers for Disease Control (CDC). Prevention and Control of Meningococcal Disease: Recommendations of the Advisory Committee on Immunization Practices (ACIP). *MMWR Recomm Rep*.

[9] Applegate A. O., Fong V. C., Tardivel K., Lippold S. A., Zarate S. (2016). Notes from the Field: Meningococcal Disease in an International Traveler on Eculizumab Therapy ? United States, 2015. *MMWR. Morbidity and Mortality Weekly Report*.

[10] Cullinan N., Gorman K. M., Riordan M., Waldron M., Goodship T. H. J., Awan A. (2015). Case report: Benefits and challenges of long-term eculizumab in atypical hemolytic uremic syndrome. *Pediatrics*.

[11] Rey-Mugica M. A. (2012). Meningococcaemia durante el tratamiento con Eculizumab. *Enferm Infec Microbol Clin*.

[12] Struijk G. H., Bouts A. H. M., Rijkers G. T., Kuin E. A. C., Ten Berge I. J. M., Bemelman F. J. (2013). Meningococcal sepsis complicating eculizumab treatment despite prior vaccination. *American Journal of Transplantation*.

[13] Alashkar F., Vance C., Herich-Terhürne D., Preising N., Dührsen U., Röth A. (2017). Serologic response to meningococcal vaccination in patients with paroxysmal nocturnal hemoglobinuria (PNH) chronically treated with the terminal complement inhibitor eculizumab. *Annals of Hematology*.

